# Supplementary material for: Effects of a mindfulness-based intervention and a health self-management programme on psychological well-being in older adults with subjective cognitive decline: Secondary analyses from the SCD-Well randomised clinical trial
Source: PLoS One. 2023 Dec 15;18(12):e0295175. doi: 10.1371/journal.pone.0295175 (PMC10723715; doi:10.1371/journal.pone.0295175)
Supplement: S1 File — (PDF) [file pone.0295175.s001.pdf]

Supporting Information

Table S1 Descriptions of the self-report measures included in the meditation-based well-being composites

The *Compassionate Love Scale* (CLS; stranger-humanity version) [1] was used to measure compassion for others. Compassion can be conceptualised as a complex response to suffering – entailing affective, behavioural, and cognitive aspects – that, importantly, includes the intention to reduce suffering. The CLS comprises 21 items with a 7-point Likert scale anchored at 1 (not at all true of me) and 7 (very true of me). Total scales scores are computed by averaging the 21 item scores. Higher total scores reflect higher levels of compassion for others. The CLS has shown high levels of internal consistency (Cronbach's alpha = 0.95) [1]; however, a recent evaluation of the CLS recommended the use of a shorter 7-item version (i.e., COS-7) [2].
The *Drexel Defusion Scale* (DDS) [3] was used to measure levels of defusion, the capacity to psychologically distance oneself from subjective experiences including body sensations, thoughts, emotions and perceptions in general. To be in a state of defusion implies that the seemingly inherent reality commonly assigned to subjective experiences is, to a certain degree, softened, thus making other ways of relating to experience more accessible (e.g. seeing sensations and thoughts as mere phenomenological events or as "just a perception"). The DDS comprises 10 items with a 6-point Likert scale ranging from 0 (not at all) to 5 (very much). Total scores are derived by summing the 10 item scores. Higher total scores reflect a greater ability to defuse from subjective experience. The DDS has displayed good psychometric properties including adequate levels of internal consistency (Cronbach's alpha = 0.95) [3].
The *39-item Five Facet Mindfulness Questionnaire* (FFMQ-39) [4] was used to measure five trait-like facets of mindfulness, namely observing (noticing experiences), describing (labelling experiences), acting with awareness (attending to activities non-mechanically), non-judging (non-evaluative stance towards experiences), and non-reactivity (allowing experiences). The FFMQ-39 comprises one 7-item scale (non-reactivity) and four 8-item scales using a 5-point Likert scale anchored at 1 (never or very rarely true) and 5 (very often or always true). After reverse scoring some items, the subscale scores are derived by summing their respective item scores. Higher subscale scores are indicative of a greater tendency to display the mindfulness facets in daily life. The FFMQ subscales have demonstrated adequate psychometric properties including good internal consistency (Cronbach's alpha ranging from 0.75 to 0.91) [4–6].
The *Multidimensional Assessment of Interoceptive Awareness* (MAIA) [7] questionnaire was used to measure eight state-trait facets of interoceptive awareness, which describe the nervous system's ability to sense, interpret, and integrate signals produced within the body. The 32-item MAIA comprises eight subscales with a 6-point Likert scale anchored at 0 (*never*) and 5 (*always*): noticing (awareness of body sensations; 4 items), not-distracting (not ignoring uncomfortable sensations; 6 items), not-worrying (not distressed by uncomfortable sensations; 5 items), attention regulation (sustaining and controlling attention on sensations; 7 items), emotional awareness (awareness of connection between sensations and emotions; 5 items), self-regulation (regulating distress by attention to sensations; 4 items), body listening (listening to the body for insight; 3 items), and trusting (experiencing the body as safe; 3 items). After reverse scoring some items, subscale scores are computed by averaging their respective item scores. Higher subscale scores are indicative of greater interoceptive awareness accessible to self-report. The MAIA subscales have displayed satisfactory to good levels of internal consistency (Cronbach's alpha ranging from 0.64 to 0.83) [7].

Table S2 Results from exploratory mixed effects models assessing differential change in PWBS dimensions

		Standardised estimated change		Difference in change CMBAS vs. HSMP	
Outcome	Time	CMBAS	HSMP	Mean (95% CI)	p
Autonomy	V1 to V2	0.22 (0.02, 0.42)	0.24 (0.03, 0.44)	0.01 (-0.22, 0.25)	0.907
	V1 to V3	0.09 (-0.10, 0.29)	0.22 (0.02, 0.41)	0.12 (-0.11, 0.36)	0.305
Environmental mastery	V1 to V2	0.01 (-0.13, 0.14)	0.03 (-0.07, 0.13)	0.02 (-0.14, 0.18)	0.784
	V1 to V3	-0.004 (-0.14, 0.13)	0.12 (-0.01, 0.26)	0.13 (-0.03, 0.28)	0.121
Personal growth	V1 to V2	0.02^{e-3} (-0.15, 0.16)	0.07 (-0.09, 0.23)	0.07 (-0.11, 0.26)	0.440
	V1 to V3	-0.02 (-0.18, 0.13)	0.05 (-0.10. 0.20)	0.07 (-0.11, 0.25)	0.438
Positive relations	V1 to V2	-0.02 (-0.15, 0.12)	-0.03 (-0.17, 0.11)	-0.01 (-0.18, 0.15)	0.875
	V1 to V3	-0.10 (-0.24, 0.04)	0.01 (-0.13, 0.14)	0.10 (-0.06, 0.27)	0.203
Self-acceptance	V1 to V2	-0.02 (-0.17, 0.12)	-0.01 (-0.16, 0.14)	0.02 (-0.16, 0.19)	0.858
	V1 to V3	0.01 (-0.13, 0.16)	0.04 (-0.11, 0.18)	0.02 (-0.15, 0.20)	0.792
Purpose in life	V1 to V2	-0.03 (-0.18, 0.12)	0.01 (-0.15, 0.16)	0.03 (-0.15, 0.21)	0.717
	V1 to V3	0.07 (-0.08, 0.22)	0.06 (-0.09, 0.20)	0.01 (-0.19, 0.16)	0.893

Note. Only participants who provided data at all three time points were included in the analyses. All analyses were adjusted for baseline scores of the outcome. PWBS = Psychological Well-being Scale; SCD = subjective cognitive decline; CI = confidence interval; CMBAS = Caring Mindfulness-based Approach for Seniors; HSMP = Health Self-Management Programme.

Table S3 Exploratory moderator analyses using linear regression models to predict change in well-being outcomes from pre- (V1) to post-intervention (V2)

Moderator	PWBS total	Psychological QoL	Awareness	Connection	Insight
CMBAS					
Session attendance	0.03 (-0.09, 0.15)	-0.02 (-0.23, 0.20)	0.01 (-0.26, 0.27)	**-0.40** (-0.65, -0.15)	0.09 (-0.19, 0.38)
Neuroticism at V1	-0.01 (-0.13, 0.11)	-0.14 (-0.34, 0.05)	-0.10 (-0.32, 0.12)	-0.05 (-0.25, 0.15)	-0.06 (-0.32, 0.20)
Outcome at V1	0.04 (-0.08, 0.16)	**-0.22** (-0.42, -0.02)	**-0.29** (-0.52, -0.07)	**-0.22** (-0.44, -4^{e-5})	**-0.28** (-0.53, -0.03)
HSMP					
Session attendance	0.02 (-0.08, 0.17)	0.21 (-0.20, 0.62)	0.19 (-0.28, 0.67)	**0.66** (0.21, 1.12)	-0.09 (-0.48, 0.29)
Neuroticism at V1	0.04 (-0.09, 0.17)	-0.17 (-0.39, 0.06)	0.15 (-0.13, 0.43)	0.16 (-0.07, 0.39)	-0.16 (-0.37, 0.05)
Outcome at V1	-0.02 (-0.14, 0.10)	-0.21 (-0.43, 0.02)	-0.15 (-0.41, 0.10)	-0.01 (-0.25, 0.23)	**-0.42** (-0.63, -0.21)

Note. All estimates are accompanied by their 95% confidence intervals. Estimates in bold were associated with $p < 0.05$. PWBS = Psychological Well-being Scale; QoL = quality of life; CMBAS = Caring Mindfulness-based Approach for Seniors; HSMP = Health Self-Management Programme.

References (Supporting information)

1. Sprecher, S. & Fehr, B. Compassionate love for close others and humanity. *J. Soc. Pers. Relatsh.* **22**, 629–651 (2005).

2. Schlosser, M., *et* al. The psychometric properties of the compassionate love scale and the validation of the English and German 7-item compassion for others scale (COS-7). *Curr. Psychol.*, 1-13 (2021).

3. Forman, E. M. *et al.* The Drexel defusion scale: A new measure of experiential distancing. *J. Context. Behav. Sci.* **1**, 55–65 (2012).

4. Baer, R. A., Smith, G. T., Hopkins, J., Krietemeyer, J. & Toney, L. Using Self-Report Assessment Methods to Explore Facets of Mindfulness. *Assessment* **13**, 27–45 (2006).

5. Baer, R. A. *et al.* Construct Validity of the Five Facet Mindfulness Questionnaire in Meditating and Nonmeditating Samples. *Assessment* **15**, 329–342 (2008).

6. Gu, J. *et al.* Examining the factor structure of the 39-item and 15-item versions of the Five Facet Mindfulness Questionnaire before and after mindfulness-based cognitive therapy for people with recurrent depression. *Psychol. Assess.* **28**, 791–802 (2016).

7. Mehling, W. E. *et al.* The Multidimensional Assessment of Interoceptive Awareness (MAIA). *PLoS One* **7**, e48230 (2012).
