# Supplementary material for: Effects of a mindfulness-based intervention and a health self-management programme on psychological well-being in older adults with subjective cognitive decline: Secondary analyses from the SCD-Well randomised clinical trial
Source: PLoS One. 2023 Dec 15;18(12):e0295175. doi: 10.1371/journal.pone.0295175 (PMC10723715; doi:10.1371/journal.pone.0295175)
Supplement: Attachment — Submitted filename: Response Letter to Reviewers.docx [file pone.0295175.s002.docx]

**Our response:** We are grateful to the reviewers and the editor for their helpful and constructive comments on our manuscript. We have carefully considered all comments, revised the manuscript accordingly, and believe that it has been significantly improved as a result. Below, we have listed our responses to each of their comments.

***Academic Editor’s Comments***

*When submitting your revision, we need you to address these additional requirements.*

*1. Please ensure that your manuscript meets PLOS ONE's style requirements, including those for file naming. The PLOS ONE style templates can be found at 
https://journals.plos.org/plosone/s/file?id=wjVg/PLOSOne_formatting_sample_main_body.pdf and 
https://journals.plos.org/plosone/s/file?id=ba62/PLOSOne_formatting_sample_title_authors_affiliations.pdf*

**Our response:** The manuscript has been adapted accordingly.

*2. Thank you for stating the following in the Competing Interests section:*

*"GC has received research support from the EU’s Horizon 2020 research and innovation programme (grant agreement number 667696), Inserm, Fondation d’entreprise MMA des Entrepreneurs du Futur, Fondation Alzheimer, Programme Hospitalier de Recherche Clinique, Région Normandie, Association France Alzheimer et maladies apparentées and Fondation Vaincre Alzheimer (all to Inserm), and personal fees from Fondation d’entreprise MMA des Entrepreneurs du Futur. All other authors have declared that no competing interests exist."*

*Please confirm that this does not alter your adherence to all PLOS ONE policies on sharing data and materials, by including the following statement: "This does not alter our adherence to  PLOS ONE policies on sharing data and materials.” (as detailed online in our guide for authors http://journals.plos.org/plosone/s/competing-interests).  If there are restrictions on sharing of data and/or materials, please state these. Please note that we cannot proceed with consideration of your article until this information has been declared.*

*Please include your updated Competing Interests statement in your cover letter; we will change the online submission form on your behalf.*

**Our response:** The updated competing interests statement has been included in the new cover letter.

*3. We note that you have indicated that data from this study are available upon request. PLOS only allows data to be available upon request if there are legal or ethical restrictions on sharing data publicly. For information on unacceptable data access restrictions, please see http://journals.plos.org/plosone/s/data-availability#loc-unacceptable-data-access-restrictions.*

*In your revised cover letter, please address the following prompts:*

*a) If there are ethical or legal restrictions on sharing a de-identified data set, please explain them in detail (e.g., data contain potentially identifying or sensitive patient information) and who has imposed them (e.g., an ethics committee). Please also provide contact information for a data access committee, ethics committee, or other institutional body to which data requests may be sent.*

*b) If there are no restrictions, please upload the minimal anonymized data set necessary to replicate your study findings as either Supporting Information files or to a stable, public repository and provide us with the relevant URLs, DOIs, or accession numbers. Please see http://www.bmj.com/content/340/bmj.c181.long for guidelines on how to de-identify and prepare clinical data for publication. For a list of acceptable repositories, please see http://journals.plos.org/plosone/s/data-availability#loc-recommended-repositories.*

*We will update your Data Availability statement on your behalf to reflect the information you provide.*

**Our response:** Our updated cover letter now addresses these prompts.

*4. One of the noted authors is a group or consortium The Medit-Ageing Research Group. In addition to naming the author group, please list the individual authors and affiliations within this group in the acknowledgments section of your manuscript. Please also indicate clearly a lead author for this group along with a contact email address.’*

*[Note: HTML markup is below. Please do not edit.]*

**Our response:** We have modified the manuscript accordingly (page 20, acknowledgments section): “Please address any correspondence relating to the Medit-Ageing Research Group to the project lead Gaël Chételat (chetelat@cyceron.fr).”

***Reviewer #1: anonymous statistical review***

*SUMMARY. This study aims to compare the effects of a mindfulness-based intervention (CMBAS) with a health self-management programme (HSMP) on mental well-being, as measured by a battery of multiple indexes. The statistical analysis correctly relies on normal mixed effects models, estimated from longitudinal data with three time points. The material is nicely organized and results are clearly described. However, I raise below some issues that should be clarified.

MAJOR ISSUES*
*1. Missing data. In this analysis missing values may arise at multiple levels. At the item level, single items could be missing. How were these missing values treated? At the occasion level, some measurements of a subject can be missing. The caption of Table 3 says: "Only participants who provided data at all three time points were included in the analyses". Why? Mixed regression models allow partial trajectories, under the assumption of a missing-at-random mechanism. Please clarify.*

**Our response:** We appreciate your concern about the handling of missing data. As now further clarified in the method section (page 11, first paragraph), our analysis adheres to the pre-registered statistical analysis plan for secondary outcomes of the Medit-Ageing Project: “In accordance with the pre-registered statistical analysis plan for secondary outcomes of the Medit-Ageing Project, in all mixed effects regression models, missing data of the well-being outcomes were not replaced and assumed to be missing-at-random.” This decision was made to ensure the consistency and reliability of our approaches across multiple publications from the Medit-Ageing Project. Instead of using mixed regression models with partial trajectories, we opted for a complete-case analysis, which we considered to be a valid and reliable method in this context (see Ross et al., 2020). Considering that our meditation composite score was used here for the first time in the literature in a longitudinal study, we evaluated a complete-case analysis to be more precise.

Reference: Ross, R. K., Breskin, A., & Westreich, D. (2020). When is a complete-case approach to missing data valid? The importance of effect-measure modification. *American journal of epidemiology*, *189*(12), 1583-1589.

*2. Covariates. Table 1 indicates that three covariates (age, gender and education) are available. It seems that they were not included in the regression models. Why? These three covariates may have a significant impact on longitudinal changes of mental well-being...*

**Our response:** We understand and appreciate your point about the potential impact of age, gender, and education on the longitudinal changes of mental well-being. However, in the interest of parsimony and based on our pre-specified hypotheses, we did not include these variables in our mixed regression models. We did not have a clear theory-based justification or prior empirical evidence to suggest that adjusting for these covariates would improve the validity of the estimated effect sizes of the interventions on well-being. While we acknowledge that a moderator analysis could assess whether these variables impact the relationship between the interventions and well-being, we did not hypothesize such a moderating effect and thus did not include them in our exploratory analyses.

*3. Normality. The implemented mixed regression models require normality of the outcomes. The authors should provide evidence that all the outcomes are normally distributed. In the case of nonnormality, either a Binomial or a Poisson assumption must be placed on the outcomes, which can be defined as the sum of multiple items. The mixed regression framework allows for such distributional extension, and it can be easily estimated by R and Stata, which are the platforms used by the authors.*

**Our response:** Mixed regression models, like many other statistical models, assume normality of the residuals, not the outcomes themselves. The residuals are the differences between the observed and predicted values in the model. The assumption of normality of residuals is important because it underpins the statistical tests that we use to determine the significance of the coefficients in the model. However, it's important to note that mixed regression models are quite robust to violations of the normality assumption (Hox et al., 2010). This means that even if the residuals are not perfectly normally distributed, the model can still provide valid results. In summary, while the assumption of normality of residuals is part of the theoretical foundation of mixed regression models, in practice, these models can handle a reasonable degree of non-normality without significant impact on the results. We ensured that our models sufficiently meet the normality assumption by visually inspecting the Q-Q plot of the residuals.

Reference: Hox, J. J., Moerbeek, M., & Van de Schoot, R. (2010). *Multilevel analysis: Techniques and applications*. Routledge.

*SPECIFIC ISSUES
1. Each outcome has been separately estimated by a single regression models with random effects. However, Table 3 does not display the estimates of the variances of the random effects. Such estimates are often not displayed when the interest is on covariate effects. However, in this work I believe that variance components could be of interest because it is possible that different outcomes are affected by different levels of latent heterogeneity.*

**Our response:** We appreciate your suggestion about the potential interest in the variances of the random effects. However, in our study, the primary focus was on the fixed effects, which directly relate to the interventions' impact on well-being. While the variance components could indeed provide additional insights into latent heterogeneity, our aim was to maintain a clear and focused presentation of the main findings. Including the variances of the random effects could potentially complicate the interpretation without substantially contributing to our primary research questions. However, we acknowledge the potential value of this information and will consider including it in future analyses where it aligns more directly with our research objectives. We believe that the current presentation of our results provides a clear and accurate representation of our findings.

*2. References are sometimes in an author-year format and other times in a numeric format. Please correct.*

**Our response:** References have been checked accordingly.

***Reviewer #2: Martin PJ van Boxtel***

*Authors acknowledge that data can be made available, but with some restrictions. I tend to agree with them, as unrestricted access to research data without input from the researchers involved may lead to erroneous conclusions. Involvement of the authors with any re-use of the data seems warranted.*

***Reviewer #3: Maggie Lawrence***

*This is a well-structured and well written paper, reporting on a topical intervention in an under-researched population. I have only a few minor comments to make:
Reference should be made to reporting guidelines e.g. CONSORT and, for example, a CONSORT diagram included.*

**Our response:** We appreciate the suggestion to include a CONSORT flow diagram in our manuscript. However, we believe it would be more efficient and less redundant to refer readers to the primary outcome paper of the SCD-Well trial, which already includes a detailed CONSORT flow diagram. This approach allows us to maintain the focus on the unique contributions of our current analysis, while still providing readers with easy access to comprehensive trial information. We trust that our readers will appreciate this streamlined approach and the effort to avoid unnecessary repetition across related publications.

*Typo: An 8-week intervention was referred to throughout the paper but on p.11 it says ‘… session attendance (out of a maximum of nine sessions) …’*

**Our response:** We thank the reviewer for highlighting this potentially confusing point. Participants actually met 9 times during this 8-week period as a one-off retreat day was included during the sixth week of the intervention. This has now been clarified in the methods section (page 11, second paragraph): “… session attendance (out of a maximum of nine sessions, i.e. 8 weekly meetings plus one retreat day), …”.

*A patient (PPI) perspective might have been useful – there is discussion around outcomes measures, but it is not reported whether these measures were perceived as being meaningful and/or relevant from the participants’ perspectives - were the trialists measuring outcomes that we meaningful to participants? And might they have had different results if patients had been involved in the selection? I’m not suggesting any change is required – I’m suggesting another point of view/discussion point – which the authors can choose to ignore!*

**Our response:** We did not measure whether the well-being outcomes were meaningful to participants. We appreciate the reviewers’ suggestion and will consider these PPI perspectives in the development of future trials.
