# Supplementary material for: Effects of a mindfulness-based intervention and a health self-management programme on psychological well-being in older adults with subjective cognitive decline: Secondary analyses from the SCD-Well randomised clinical trial
Source: PLoS One. 2023 Dec 15;18(12):e0295175. doi: 10.1371/journal.pone.0295175 (PMC10723715; doi:10.1371/journal.pone.0295175)
Supplement: Attachment — Submitted filename: renamed_e64c2.docx [file pone.0295175.s003.docx]

# COMMENTS – Manuscript PONE-D-23-18446

Title: “Effects of a mindfulness-based intervention and a health self-management programme on psychological well-being in older adults with subjective cognitive decline: Secondary analyses from the SCD-Well randomised clinical trial”

**Important note: This review pertains only to ‘statistical aspects’ of the study and so ‘clinical aspects’ [like medical importance, relevance of the study, ‘clinical significance and implication(s)’ of the whole study, etc.] are to be evaluated [should be assessed] separately/independently. Further please note that any ‘statistical review’ is generally done under the assumption that (such) study specific methodological [as well as execution] issues are perfectly taken care of by the investigator(s). This review is not an exception to that and so does not cover clinical aspects {however, seldom comments are made only if those issues are intimately / scientifically related & intermingle with ‘statistical aspects’ of the study}. Agreed that ‘statistical methods’ are used as just tools here, however, they are vital part of methodology [and so should be given due importance]. I look at the manuscript in/with statistical view point, other reviewer(s) look(s) at it with different angle so that in totality the review is very comprehensive. However, there should be efforts from authors side to improve (may be by taking clues from reviewer’s comments). Therefore, please do not limit the revision only (with respect) to comments made here.**

**COMMENTS:** Although this manuscript is well drafted and the study seems to have enough potential (is on very important/useful topic), I have few doubts/observations/concerns (different opinion) which are little serious and are given below:

In ‘Methods’ section it is stated that ‘Detailed information about the recruitment procedures, eligibility criteria, design of the interventions, and assessments can be found in the trial protocol’ and quoted reference (18). But reference 18 seems to be a report of the trial and not the ‘PROTOCOL’. It is stated there that ‘The treatment is based on a published manual [24], with every session of the program covering different subjects, including self-management’ which indicates that some sort of / some amount of ‘self-management’ component was there. Then is it not necessary to clarify regarding the overlap? It is well-known (NET/WWW search) that “Secondary analysis refers to the use of existing research data to find answer to a question that was different from the original work” then is this study can be called as ‘**Secondary analyses**’ [as mentioned in title]? I am sure that the authors are aware of limitations/disadvantages of Secondary Data Analysis, however, I request authors to kindly read the following pasted from one famous standard textbook on ‘Medical Research Methodology’:

Since the researcher did not collect the data, he or she has no control over what is contained in the data set. Often times this can limit the analysis or alter the original questions the researcher sought out to answer.

You may consider to change the ‘title’ (only if convinienced). The present study (manuscript) seems to be a report of component/part [hitherto unpublished in this form] of the same (the SCD-Well randomised controlled trial of the European) trial. Further, please note that though application of ‘Mixed effects models’ here is perfectly alright, they are [any regression techniques for that matter] are not basically/originally developed for any sort of [between or within group(s)] comparison(s). In ‘Methods’ section you stated that “Mixed effects models were used to assess between- and within-group differences in change”. Head-to-head comparison is expected, as this is [through ‘Mixed effects models’] an indirect/secondary/by-product testing, in my opinion. Application of ‘Mixed effects models’ could definitely be (useful) addition (I am sure).

Note that, though the measures/tools used are appropriate {example: 42-item *Psychological Well-being Scale* (PWBS), *Quality of Life Assessment (by WHOQOL-BREF),* four self-report measures (displayed in Table 2) - The *Multidimensional Assessment of Interoceptive Awareness* (MAIA) questionnaire and the *39-item Five Facet Mindfulness Questionnaire* (FFMQ-39), etc.}, most of them are likely to yield data that are in [at the most] ‘ordinal’ level of measurement [and not in ratio level of measurement for sure {as the score two times higher does not indicate presence of that parameter/phenomenon as double (for example, a Visual Analogue Scales VAS score or say ‘depression’ score)}]. Then application of suitable non-parametric (or distribution free) test(s) is/are indicated/advisable [even if distribution may be ‘Gaussian’ (also called ‘normal’)]. Agreed that there is/are no non-parametric test(s)/technique(s) available to be used as alternative in all situation(s), but should be used whenever/wherever they are available. Therefore, in short use suitable non-parametric test(s)/technique(s) while dealing with data that are in ‘ordinal’ level of measurement even if [despite that] the distribution may be ‘Gaussian’. Testing ‘normality’ in sample [by using any normality test(s)} is not required/desired while dealing with data that are in ‘ordinal’ level of measurement [as most of the normality tests are not valid for ‘ordinal’ data].

It may also (as often said) please be noted, [as you stated: Each dimension is measured by a 7-item subscale using a 7-point Likert scale ranging from 1 (strongly agree) to 7 (strongly disagree). After reverse scoring 21 items, subscale scores were derived by averaging their respective item scores; a total score was derived by averaging all items] that whenever response options ranged from 1 (=strongly agree) to 7 (=strongly disagree) {or from 1=very bad to 3=neither good nor bad to 5=very good), while using a ‘Likert’ scale responses, recoding [like strongly disagree=-2, disagree=-1, neutral=0, agree=1, strongly agree=2] may yield correct and meaningful ‘arithmetic mean’ which is useful not only for comparison but has absolute meaning, in my opinion. Application of any statistical test(s) assume that meaning of entity used (mean, SD, etc) has a particular meaning. Though ‘α’ [alpha] or most other measures of reliability/correlation will remain same, however, use of non-parametric methods should/may be preferred while dealing with data yielded by any questionnaire/score.

At the end of ‘Introduction’ section it is said that “We hypothesised that both interventions would improve well-being but that CMBAS would be superior to HSMP”, then what about the principle of ‘equipoise’ [equipoise means that there is genuine uncertainty in the expert medical community over whether a treatment will be beneficial. An ethical dilemma arises in a clinical trial when the investigator(s) begin to believe that the treatment or intervention administered in one arm of the trial is significantly outperforming the other arms.]? Again, as often said, some sort of ‘bias’ is (are) likely be introduced/present when the principle of ‘equipoise’ is not observed/followed.

In ‘Statistical analyses -Sample size’ section is it adequate to say “Sample size calculations in SCD-Well were based on the expected effect size ….’? Do not it necessary to mention ‘what that expected effect size’ you are referring to?

Except these minor points, the article is acceptable [drafting is excellent]. Nevertheless, mind you that as pointed out in ‘important note’ above “This review pertains only to ‘statistical aspects’ of the study and so ‘clinical aspects’ should be assessed separately/independently. ‘Minor Revision’ is recommended.
