# Supplementary material for: Effects of a mindfulness-based intervention and a health self-management programme on psychological well-being in older adults with subjective cognitive decline: Secondary analyses from the SCD-Well randomised clinical trial
Source: PLoS One. 2023 Dec 15;18(12):e0295175. doi: 10.1371/journal.pone.0295175 (PMC10723715; doi:10.1371/journal.pone.0295175)
Supplement: Attachment — Submitted filename: Response Letter to Reviewers.docx [file pone.0295175.s004.docx]

**Our response:** We are grateful to the reviewers and the editor for their helpful and constructive comments on our manuscript. We have carefully considered all comments, revised the manuscript accordingly, and believe that it has been significantly improved as a result. Below, we have listed our responses to each of their comments.

***Academic Editor’s Comments***

*1. Please ensure that your manuscript meets PLOS ONE's style requirements, including those for file naming. The PLOS ONE style templates can be found at*

*https://journals.plos.org/plosone/s/file?id=wjVg/PLOSOne_formatting_sample_main_body.pdf and*

[*https://journals.plos.org/plosone/s/file?id=ba62/PLOSOne_formatting_sample_title_authors_affiliations.pdf*](https://journals.plos.org/plosone/s/file?id=ba62/PLOSOne_formatting_sample_title_authors_affiliations.pdf)

**Our response:** The manuscript has been modified accordingly.

*2. Thank you for stating the following in the Competing Interests section:*

*“GC has received research support from the EU’s Horizon 2020 research and innovation programme (grant agreement number 667696), Inserm, Fondation d’entreprise MMA des Entrepreneurs du Futur, Fondation Alzheimer, Programme Hospitalier de Recherche Clinique, Région Normandie, Association France Alzheimer et maladies apparentées and Fondation Vaincre Alzheimer (all to Inserm), and personal fees from Fondation d’entreprise MMA des Entrepreneurs du Futur. All other authors have declared that no competing interests exist.”*

*Please confirm that this does not alter your adherence to all PLOS ONE policies on sharing data and materials, by including the following statement: "This does not alter our adherence to  PLOS ONE policies on sharing data and materials.” (as detailed online in our guide for authors http://journals.plos.org/plosone/s/competing-interests).  If there are restrictions on sharing of data and/or materials, please state these. Please note that we cannot proceed with consideration of your article until this information has been declared.*

*Please include your updated Competing Interests statement in your cover letter; we will change the online submission form on your behalf.*

**Our response:** The updated Competing Interests statement has been included in the revised cover letter.

*3. One of the noted authors is a group or consortium [The Medit-Ageing Research Group]. In addition to naming the author group, please list the individual authors and affiliations within this group in the acknowledgments section of your manuscript. Please also indicate clearly a lead author for this group along with a contact email address.*

**Our response:** The acknowledgments section has been revised accordingly (page 20): “Please address any correspondence relating to the Medit-Ageing Research Group to the project lead Gaël Chételat ([chetelat@cyceron.fr)](mailto:chetelat@cyceron.fr)).”

***Reviewer #1***

*Although this manuscript is well drafted and the study seems to have enough potential (is on very important/useful topic), I have few doubts/observations/concerns (different opinion) which are little serious and are given below:*

*1. In ‘Methods’ section it is stated that ‘Detailed information about the recruitment procedures, eligibility criteria, design of the interventions, and assessments can be found in the trial protocol’ and quoted reference (18). But reference 18 seems to be a report of the trial and not the ‘PROTOCOL’. It is stated there that ‘The treatment is based on a published manual [24], with every session of the program covering different subjects, including self-management’ which indicates that some sort of / some amount of ‘self-management’ component was there. Then is it not necessary to clarify regarding the overlap?*

**Our response:** We thank the reviewer for encouraging us to clarify the difference between the interventions. Both CMBAS and HSMP included self-management components and overlap in format and structure, which are described on page 7 (last paragraph) and page 8 (second paragraph): “CMBAS also included psychoeducational components that offered participants approaches to deal with cognitive concerns and tendencies to worry in skilful ways.” & “HSMP … included guidance on exercise, stress, memory, communication, healthy eating, and the management of sleep.” The citation to the published manual on which HSMP was based has now been included.

*2. It is well-known (NET/WWW search) that “Secondary analysis refers to the use of existing research data to find answer to a question that was different from the original work” then is this study can be called as ‘Secondary analyses’ [as mentioned in title]? I am sure that the authors are aware of limitations/disadvantages of Secondary Data Analysis, however, I request authors to kindly read the following pasted from one famous standard textbook on ‘Medical Research Methodology’:
Since the researcher did not collect the data, he or she has no control over what is contained in the data set. Often times this can limit the analysis or alter the original questions the researcher sought out to answer. You may consider to change the ‘title’ (only if convinienced).*

**Our response:** We thank the reviewer for asking us to clarify this important point. The current manuscript presents data from the European Medit-Ageing Project. The authors have substantially contributed to the design of the Medit-Ageing Project or the acquisition, analysis, or interpretation of its data.

*3. The present study (manuscript) seems to be a report of component/part [hitherto unpublished in this form] of the same (the SCD-Well randomised controlled trial of the European) trial.*

**Our response:** This is correct. The current manuscript presents secondary analyses from the SCD-Well trial, which the authors have conducted. The primary outcome paper of this trial has already been published (page 6, fourth paragraph): “The primary outcome of the SCD-Well trial was mean change in anxiety symptoms from V1 to V2 (9).”

*4. Further, please note that though application of ‘Mixed effects models’ here is perfectly alright, they are [any regression techniques for that matter] are not basically/originally developed for any sort of [between or within group(s)] comparison(s). In ‘Methods’ section you stated that “Mixed effects models were used to assess between- and within-group differences in change”. Head-to-head comparison is expected, as this is [through ‘Mixed effects models’] an indirect/secondary/by-product testing, in my opinion. Application of ‘Mixed effects models’ could definitely be (useful) addition (I am sure). Even if ultimate results are same, one should follow a correct way (I think).*

**Our response:** We agree with the reviewer that the mixed effects models we have employed are an appropriate and effective method to answer our research questions.

*5. Note that, though the measures/tools used are appropriate {example: 42-item Psychological Well-being Scale (PWBS), Quality of Life Assessment (by WHOQOL-BREF), four self-report measures (displayed in Table 2) - The Multidimensional Assessment of Interoceptive Awareness (MAIA) questionnaire and the 39-item Five Facet Mindfulness Questionnaire (FFMQ-39), etc.}, most of them are likely to yield data that are in [at the most] ‘ordinal’ level of measurement [and not in ratio level of measurement for sure {as the score two times higher does not indicate presence of that parameter/phenomenon as double (for example, a Visual Analogue Scales VAS score or say ‘depression’ score)}]. Then application of suitable non-parametric (or distribution free) test(s) is/are indicated/advisable [even if distribution may be ‘Gaussian’ (also called ‘normal’)]. Agreed that there is/are no non-parametric test(s)/technique(s) available to be used as alternative in all situation(s), but should be used whenever/wherever they are available. Therefore, in short use suitable non-parametric test(s)/technique(s) while dealing with data that are in ‘ordinal’ level of measurement even if [despite that] the distribution may be ‘Gaussian’. Testing ‘normality’ in sample [by using any normality test(s)} is not required/desired while dealing with data that are in ‘ordinal’ level of measurement [as most of the normality tests are not valid for ‘ordinal’ data].*

**Our response:** We used standardised, validated self-report measures that are widely used. The present study is replicating the methodology previously used in Schlosser et al. 2021. We have scored these measures based on the standardised scoring procedure presented in the development and validation papers of the respective scales. Citations of these development and validation papers are included in our manuscript. We confirmed that the distribution of these measures are supporting the assumptions required for using mixed effects models.

*6. It may also (as often said) please be noted, [as you stated: Each dimension is measured by a 7-item subscale using a 7-point Likert scale ranging from 1 (strongly agree) to 7 (strongly disagree). After reverse scoring 21 items, subscale scores were derived by averaging their respective item scores; a total score was derived by averaging all items] that whenever response options ranged from 1 (=strongly agree) to 7 (=strongly disagree) {or from 1=very bad to 3=neither good nor bad to 5=very good), while using a ‘Likert’ scale responses, recoding [like strongly disagree=-2, disagree=-1, neutral=0, agree=1, strongly agree=2] may yield correct and meaningful ‘arithmetic mean’ which is useful not only for comparison but has absolute meaning, in my opinion. Application of any statistical test(s) assume that meaning of entity used (mean, SD, etc) has a particular meaning. Though ‘α’ [alpha] or most other measures of reliability/correlation will remain same, however, use of non-parametric methods should/may be preferred while dealing with data yielded by any questionnaire/score.*

**Our response:** We agree that the self-report measures we utilised produced meaningful scores and that these scores were appropriately analysed.

*7. At the end of ‘Introduction’ section it is said that “We hypothesised that both interventions would improve well-being but that CMBAS would be superior to HSMP”, then what about the principle of ‘equipoise’ [equipoise means that there is genuine uncertainty in the expert medical community over whether a treatment will be beneficial. An ethical dilemma arises in a clinical trial when the investigator(s) begin to believe that the treatment or intervention administered in one arm of the trial is significantly outperforming the other arms.]? Again, as often said, some sort of ‘bias’ is (are) likely be introduced/present when the principle of ‘equipoise’ is not observed/followed.*

**Our response:** We believe it is common practice to develop theoretically grounded predictions about the potentially differential effects of two different interventions on a particular outcome, as we described on page 6 (second paragraph): “We hypothesised that both interventions would improve well-being but that CMBAS would be superior to HSMP, because, based on previous research and theory (7,16,17), we predicted embodied meditative practices aimed at deep human flourishing to be a more potent catalyst of well-being than health educational instructions.”

*8. In ‘Statistical analyses -Sample size’ section is it adequate to say “Sample size calculations in SCD-Well were based on the expected effect size ….’? Do not it necessary to mention ‘what that expected effect size’ you are referring to?*

**Our response:** We thank the reviewer for highlighting this point, which we have now clarified (page 10, last paragraph): “Sample size calculations in SCD-Well were based on the expected effect size (0.5, based on a meta-analysis of the efficacy of meditation-based interventions for reducing anxiety symptoms; (25)) with 80% power and two-sided type I error of 5% for the mean change in trait-STAI scores from V1 to V2 between CMBAS and HSMP, resulting in a minimum total number of 128 (64 per group), which has been exceeded (n = 147; detailed in (18).”

*9. Except these minor points, the article is acceptable [drafting is excellent]. Nevertheless, mind you that as pointed out in ‘important note’ above “This review pertains only to ‘statistical aspects’ of the study and so ‘clinical aspects’ should be assessed separately/independently. ‘Minor Revision’ is recommended.*

***Reviewer #2***

*This is a well-written manuscript reporting the findings of an RCT comparing the effects of a mindfulness-based intervention vs. a health education program on wellbeing-related outcomes in participants with subjective cognitive decline. I will defer to the statistical reviewer regarding the appropriateness and rigor of the statistical analysis. While the data is not being made fully available without restriction, it appears that the authors have addressed the issue satisfactorily. The following are items I believe still need to be addressed.*

*1. It appears that some items recommended by CONSORT were not reported. I suggest consulting the CONSORT NPT extension and addressing any items that are currently lacking. I believe PLOS ONE also requires a completed CONSORT checklist and flow diagram as part of the submission for manuscripts reporting results of clinical trials.*

**Our response:** We thank the reviewer for prompting us to revisit the Plos One requirements, which our manuscript now aligns with. We appreciate the suggestion to include a CONSORT flow diagram in our manuscript. However, we believe it would be more efficient and less redundant to refer readers to the primary outcome paper of the SCD-Well trial, which already includes a detailed CONSORT flow diagram. This approach allows us to maintain the focus on the unique contributions of our current analysis, while still providing readers with easy access to comprehensive trial information. We trust that our readers will appreciate this streamlined approach and the effort to avoid unnecessary repetition across related publications.

*2. The citation style is inconsistent and should be corrected.*

**Our response:** This has been corrected.

*3. In describing the HSMP intervention a published manual is mentioned. A citation to the manual should be provided.*

**Our response:** This citation has now been included:

Lorig K, Holman H, Sobel D, Laurent D, Gonzalez V, Minor M. Living a Healthy Life with Chronic Conditions: For Ongoing Physical and Mental Health Conditions. 4th ed. Bull Publishing Company; 2012.

*4. In the comparative analyses section a pre-registered statistical analysis plan is mentioned. If this plan is available, information on where to access it should be provided.*

**Our response:** We thank the reviewer for noting this omission. This has now been clarified (page 11, first paragraph): “The data and analysis plan underlying this paper are made available on request following approval by the executive committee and a formal data sharing agreement (https://silversantestudy.eu/2020/09/25/data-sharing).”

*5. Session attendance is included as a potential moderator variable, yet home practice did not seem to be included. Given that the majority of the time spent doing the intervention appears to be during home practice, I believe it warrants attention. On a related note, information on adherence (e.g., number of sessions attended, number/hours of home practice completed) would also be useful.*

**Our response:** We are grateful for the encouragement to include this additional data in the manuscript. Information regarding adherence has now been added (page 12, first paragraph): “There were no significant differences between the interventions for the mean number of sessions attended (CMBAS = 6.7; HSMP = 6.8), the proportion of participants who attended at least four sessions (CMBAS = 81%; HSMP = 85%), or the proportion of participants who reported continued engagement with intervention activities between V2 and V3 (CMBAS = 59%; HSMP = 54%). There were no significant differences between the proportions of participants who completed home practice on at least four occasions (CMBAS = 55 [75%]; HSMP = 51 [69%]).”

Home practice was not included as a moderator as the home practice assigned to participants differed between CMBAS and HSMP (reflecting the interventions’ distinct rationales and themes). For CMBAS, the home practices were relatively fixed and prescribed by the facilitator, whereas participants in HSMP devised their own action plans based on their own goals. Improving the similarity of home practice assignments across intervention arms will be an important consideration for future trials.

*6. Looking at table 2, in general it appears there was a drop off in the number of participants from V1 to V2 for both groups, then V3 rebounded a bit for the HSMP group but not the CMBAS group. Was there some reason that might explain this difference?*

**Our response:** There was no systematic reason for this difference. Some participants in the HSMP group were unavailable to attend their V2 visit for personal reasons.

*7. When discussing generalizability of the findings, it is mentioned that the participants were largely white. The data supporting this should be provided in Table 1.*

**Our response:** This information has now been added to Table 1.
