# Supplementary material for: Effects of a mindfulness-based intervention and a health self-management programme on psychological well-being in older adults with subjective cognitive decline: Secondary analyses from the SCD-Well randomised clinical trial
Source: PLoS One. 2023 Dec 15;18(12):e0295175. doi: 10.1371/journal.pone.0295175 (PMC10723715; doi:10.1371/journal.pone.0295175)
Supplement: Attachment — Submitted filename: Response Letter to Reviewers.docx [file pone.0295175.s005.docx]

**Our response:** We are grateful to the reviewers for their additional comments on our manuscript. We have considered all comments and revised the manuscript accordingly. Below, we have listed our responses to each of their comments.

***Reviewer #1***

*All the comments are answered and so I recommend the acceptance because the manuscript has now achieved the acceptable level.
[However, please note that I suggested (refer to item 4) “Head-to-head comparison” {and said comparative inference drawn through ‘Mixed effects models’ done here is an indirect/secondary/by-product testing, in my opinion. Application of ‘Mixed effects models’ could definitely be (useful) addition (I am sure). Even if ultimate results are same, one should follow a correct way (I think)]. In response it is said that “We agree with the reviewer that the mixed effects models we have employed are an appropriate and effective method to answer our research questions” and there is NO mention of “Head-to-head comparison”.]*

**Our response:** Thank you for your comment on the application of mixed effects models and for your prior suggestions regarding the statistical aspects of our manuscript. We believe that the implementation of your feedback has substantially improved our manuscript. Regarding mixed models, we believe that our application of these models aligns with current best practices in the field and provides a rigorous assessment of our hypotheses. We agree that alternative statistical approaches could have been employed to analyse the data. In the interest of parsimony, head-to-head comparisons of our mixed effects models with other alternative statistical approaches for analysing the data were not performed.

***Reviewer #3***

*In the introduction, the authors indicated “Although the condition could
be an indication of prodromal Alzheimer’s disease (AD), which is the most common form of
dementia (3), SCD has also been related to other factors (e.g., physical and mental illness,
sleep disturbances, personality traits, effects of drugs).” It could be that the lack of effect is due to these other factors, especially when there is no consensus of what constitutes an SCD. It will be prudent to address this issue in the discussion.*

**Our response:** We thank the reviewer for this valuable suggestion. The following sentences has now been added to the discussion section (page 17, last paragraph): “Further, the limited intervention effects could also be related to factors that have been associated with SCD but were not sufficiently captured in the context of the present study (e.g., sleep disturbances measured by polysomnography). Longitudinal studies with longer training periods and additional measures of physical and mental health are needed to further elucidate these questions and other potential dose-response relationships between meditation practice and diverse aspects of psychological well-being in older adults.”
